# Endometritis and endometrial fibrosis are associated with alterations in mare endometrial proteome

**DOI:** 10.1186/s12917-026-05691-6

**Published:** 2026-07-08

**Authors:** Mariana Leal, Elisabete Silva, Joana Alpoim-Moreira, Pedro Pinto-Bravo, Maria Rosa Rebordão, Miguel Quaresma, Beenu Jalali, Tomasz Molcan, Anna Szóstek-Mioduchowska, Dariusz Skarzynski, Graça Ferreira-Dias

**Affiliations:** 1 Faculty of Veterinary Medicine, CIISA, University of Lisbon, Lisbon, Portugal; 2 Associate Laboratory for Animal and Veterinary Sciences (AL4AnimalS), Lisbon, Portugal; 3 Faculty of Veterinary Medicine, Lusófona University, Lisbon, Portugal; 4 Polytechnic University of Coimbra, Coimbra Agriculture School, Bencanta, Coimbra, 3045-601 Portugal; 5 Research Center for Natural Resources, Environment (CERNAS), Polytechnic University of Coimbra, Bencanta, Coimbra, 3045-601 Portugal; 6 Animal and Veterinary Research Center (CECAV), University of Trás-os-Montes e Alto Douro (UTAD), Quinta de Prados, Vila Real, 5000-801 Portugal; 7 Institute of Animal Reproduction and Food Research Polish Academy of Sciences, Olsztyn, 10-643 Poland; 8 Faculty of Veterinary Medicine, University of Environmental and Live Sciences, Wroclaw, Poland

**Keywords:** Endometritis, Endometrial fibrosis, Mare, Endometrial proteome, Inflammation

## Abstract

Equine persistent post-breeding endometritis may lead to endometrosis, characterized by excessive fibrosis, which is a major cause of subfertility/infertility. Endometrial proteomic changes induced by endometritis, when associated with fibrosis, were investigated to identify the biological processes (BP) involved. Cyclic mares´ endometrial biopsies were histologically classified (Kenney and Doig’s) as: category IIA (*n* = 5), category IIA with endometritis (IIA-E; *n* = 4), category IIB (*n* = 5), or category IIB with endometritis (IIB-E; *n* = 5). Proteome was assessed by LC-MS/MS. In total, 62 differentially abundant proteins (DAPs) were identified in IIA-E endometria, compared to IIA; and 46 in IIB-E endometria, compared to IIB. The DAPs in endometria with endometritis (IIA-E vs. IIA; IIB-E vs. IIB) were mostly enriched in BP related to immune/inflammatory response, DNA-related process and cell cycle. Inflammatory-related proteins (serpin proteins and several phospholipases) were upregulated in endometritis, regardless of the degree of fibrosis and inflammation, suggesting the activation of conserved innate immune mechanisms. Nevertheless, fibrosis severity determined the expression pattern of histone H2A, which was upregulated in IIA-E endometria, and downregulated in IIB-E endometria. Additionally, other inflammatory-related proteins (azurocidin 1, eosinophil peroxidase and secreted phosphoprotein 1) were differently abundant only in endometritis associated with endometrosis (IIB-E), indicating that moderate fibrosis and endometrial degeneration may influence the inflammatory and tissue remodelling response. In conclusion, although conserved innate immune mechanisms operate independently of fibrosis severity and of endometrial inflammatory state, other processes activated during endometritis are influenced by the endometrium milieu, namely by the progression of endometrial fibrosis, which might modulate specific molecular responses.

**Supplementary Information:**

The online version contains supplementary material available at https://doi.org/10.1186/s12917-026-05691-6.

## Introduction

 Endometritis and endometrial fibrosis are the most common causes of subfertility or infertility in mares, resulting in significant economic losses. Endometritis reduces conception rates and increases the risk of several complications, including early embryonic death and abortion [1]. In mares, transient breeding-induced endometritis is a physiological mechanism in which the inflammatory response is required for the effective removal of debris, bacteria and non-viable sperm from the uterine lumen [1–3]. Inflammation activates the innate and humoral immune responses which, together with the physical clearance of uterine contents, restore homeostasis. Impaired uterine clearance, caused by impaired myometrial activity, leads to fluid accumulation and an inflammatory response, and appears to be the key factor in the pathogenesis of persistent endometritis [1, 2].

Mares differ in their susceptibility to endometritis. A resistant mare is able to clear uterine inflammation within 48 h, while a susceptible mare remains affected [2–4]. In fact, around 15% of mares are unable to clear this transient inflammation and develop persistent breeding-induced endometritis [4, 5]. The inflamed uterine milieu is not only incompatible with the survival of the embryo by the time it descends into the uterine lumen on day 6 of pregnancy [6], but also predisposes to the proliferation of pathogenic or opportunistic microorganisms, thereby increasing the risk of infection. Chronic endometritis in the mare is mainly associated with bacterial infections, although it can also be caused by fungi [4]. The most frequently isolated bacteria are *Escherichia coli*,* Streptococcus equi*,* Pseudomonas aeruginosa* and *Staphylococcus aureus* [2, 4, 5]. The diagnosis is often made by ultrasound, to detect the presence of uterine fluid, followed by endometrial cytology and microbiological culture. An endometrial biopsy may also be required [2].

Chronic endometritis may lead to endometrosis, characterized by severe fibrosis accompanied by cystic dilatation of glands in the endometrium [4, 7]. Endometrial fibrosis is an irreversible condition that tends to aggravate with age [8]. Its definite diagnosis can only be achieved by histological evaluation of an endometrial biopsy. A system for endometrial biopsy classification, developed by Kenney and Doig, classifies the endometrium into 4 categories (I, IIA, IIB and III), based on the degrees of severity of inflammatory and fibrotic changes and the prognosis for fertility in mares [7, 9]. Fertility decreases according to the severity of these changes and may drop to 10% in mares with severe inflammation and extensive fibrosis (category III) [10]. While category III mares are mostly infertile, those in category IIA and IIB may still benefit from appropriate clinical care to improve pregnancy outcomes.

A few studies have compared the proteome of uterine fluid from healthy mares with mares with endometritis [5, 11, 12]. These studies showed that most of the proteins detected in the uterine fluid of healthy mares were related to endometrial remodelling and early embryonic development, while proteins related to the generation of energy and REDOX (reduction–oxidation) regulation, neutrophil extracellular trap formation, and innate immune and inflammatory responses were upregulated in the uterine fluid of mares with infectious endometritis. Proteomic studies on equine endometrial tissue have the potential to identify less abundant proteins that are not detected in the uterine fluid but are-relevant to this equine uterine disease.

The present study hypothesized that the uterine response to endometritis is influenced by the degree of endometrial fibrosis. Therefore, this work aimed to compare mare endometrial proteome from biopsies categorized as IIA and IIB (Kenney and Doig’s classification), with and without endometritis, to investigate whether proteomic changes induced by endometritis differ according to the severity of endometrial fibrosis.

## Materials and methods

### Animals and samples

A total of 19 cyclic breed mares, aged between 3 and 19 years old, from different stud farms located in Portugal were included in this study (February to July), during the reproductive season. This study was carried out in the context of the reproductive management of the stud farms. Before sampling, each mare was submitted to a clinical examination to exclude other health issues. Ultrasound examination of internal genitalia was performed every other day. Endometrial double-guarded swabs were taken and the samples analyzed for cytology and microbiology. For endometritis diagnosis, the uterine fluid accumulation was evaluated by ultrasound. One endometrial biopsy per mare was retrieved with a biopsy alligator jaw forceps (141965, Kruuse, Denmark). Tissue was divided into two parts; one piece was placed in 4% buffered formaldehyde solution for histologic analysis, and the other piece was immersed in RNAlater (AM7021, Thermo Fisher Scientific, Lithuania) for protein isolation, kept for 24 h at 4 °C, and then stored at -80 °C. Formaldehyde-fixed endometrium was embedded in paraffin, and 4 μm-thick histological sections stained with haematoxylin (05-06014E, Bio-Optica, Italy) and eosin (HT1103128, Sigma-Aldrich, USA), and observed by light microscopy (Leica DM500) at 400X magnification.

Endometrial biopsies were classified according to Kenney and Doig (1986). Only biopsies classified as category IIA or IIB, with or without endometritis, were selected for this study. These categories are frequently associated with subfertility and represent clinically relevant cases in equine reproductive practice, which require clinicians care to get in foal. Endometria with mild fibrosis (with 1–3 layers of fibroblasts surrounding glands or less than 2 fibrotic nests per 5 mm linear field), either with or without mild scattered inflammation, were considered as category IIA. Endometria with moderate fibrosis (with 4 or more layers of fibroblasts surrounding the endometrial glands or 2–4 fibrotic nests per 5 mm linear field), either with or without mild scattered inflammation, were graded as category IIB (moderate endometrosis) [9].

Mares were classified as having endometritis when they have history of infertility and/or irregular estrous cycles, and at least three of the following diagnostic criteria were present: (1) intra-uterine fluid detected by ultrasonography (> 48 h post-breeding), (2) excessive endometrial fold edema, (3) positive endometrial cytology, (4) positive bacterial culture, and (5) histopathological evidence of endometrial inflammation [13]. Cytology was considered positive for inflammation in the presence of more than two neutrophils per four microscopic fields/high power field over 10 fields in endometrial cytology (mag = 400X) [2]. Microbiology culture was considered positive when one or two bacterial species were isolated (contamination was considered when three or more species grew in culture) [2].

Due to the clinical context of sample collection, mares were biopsied at different stages of the estrous cycle, and samples from both estrus and diestrus were included in each endometrial category. However, principal component analysis (PCA) within each endometrial category did not reveal clustering by cycle stage (estrus vs. diestrus), suggesting that the pathological status of the endometrium had a greater impact on the proteomic profile than the estrous cycle phase.

Endometrial biopsies were assigned to four groups: category IIA without endometritis (*n* = 5; aged between 3 and 9 years), category IIA with endometritis (IIA-E) (*n* = 4; aged between 5 and 19 years), category IIB without endometritis (*n* = 5; aged between 6 and 17 years) and category IIB with endometritis (IIB-E) (*n* = 5; aged between 9 and 18 years).

Procedures were performed by a veterinarian as part of a breeding examination at the request of the mare’s owner, who was fully informed and gave informed consent for the use of the data for research purposes.

### Total protein extraction

Protein extraction was performed using 30 mg of endometrial tissue. Tissues were lysed in Tissue Protein Extraction Reagent (Ref.78510, Thermo Fisher Scientific, Lithuania) containing protease inhibitor (A32953, Thermo Fisher Scientific, Lithuania) and disrupted using the Tissue Lyser (Qiagen), followed by incubation on ice for 30 min, and centrifugation. The protein concentration was quantified by the Bradford method (1856209, Thermo Fisher Scientific, Lithuania).

### Proteomic analysis

#### LC-MS/MS analysis and protein validation

Samples were run in triplicate (50 µg) and reduced with dithiothreitol (Sigma) (10mM, 45 min, 56 °C), alkylated with iodoacetamide (Sigma) (20mM, 30 min, in the dark) and precipitated with acetone. After drying at room temperature, precipitates were solubilized in ammonium bicarbonate (50mM) and digested with trypsin (Promega) (1:50, overnight, 37 °C). Digestion was stopped with formic acid (0.5%) and the peptides were purified using OMIX C18 microcolumns (Agilent Technologies), lyophilized and resuspended in 0.1% solution of formic acid in water (Buffer A). Liquid chromatography-tandem mass spectrometry (LC-MS/MS) analysis was performed on a Waters M-Class HPLC (Waters, Massachusetts) coupled to a ZenoTOF 7600 system (SCIEX, Redwood City, California) with an OptiFlow Turbo V Ion Source using a micro electrode (1–10 µL/min). Peptides were separated in direct injection mode (5 µL/min) on a Kinetex XB-C18 analytical column (150 × 0.30 mm, 2.6-µm particle size; Phenomenex), with an acetonitrile/FA gradient. Samples were analyzed by sequential window acquisition of all theoretical fragment ion spectra (SWATH)-MS. SWATH-MS data were acquired in SWATH acquisition mode using a set of 85 overlapping variable SWATH windows (350–1,250 m/z) covering the precursor mass.

Data processing was performed using DIA-NN (version 1.8.1) library free workflow. The reference proteome for *Equus caballus* (Uniprot ID UP000002281) was converted to a theoretical spectral ion library file. SWATH data was then analyzed using DIA-NN set as follows: precursor FDR 1%; mass accuracy at MS1 and MS2 set to 0 ppm; scan window set to 0; isotopologues and MBR turned on; protein inference at gene level; heuristic protein inference enabled; quantification strategy set to Robust LC (high precision); neural network classifier single-pass mode; cross-run normalization RT-dependent; MBR enable. In the library-free mode, the main search settings were the same with additional settings for in silico library generation as follows: Trypsin/P with maximum 1 missed cleavage; protein N-terminal M excision on; Carbamidomethyl on C as fixed modification; no variable modification; peptide length from 7 to 30; precursor charge 1–4; precursor m/z from 350 to 1250; fragment m/z from 100 to 1500. Data were directly exported to Markerview 1.3.1 (Sciex, Framingham, MA USA) and normalized using total area sums to obtain the final quantification values.

The mass spectrometry proteomics data have been deposited to the ProteomeXchange Consortium via the PRIDE [14, 15] partner repository with the dataset identifier PXD070769.

#### Proteomic data analysis

Data analysis included only proteins with valid quantification values (LFQ) in at least three biological replicates, and two or more unique peptides per replicate. Differentially abundant proteins (DAPs) were statistically tested using the student’s t-test, considering a *P*-value < 0.001 and a log2 Fold Change ≥ 1 or a Log2 Fold Change ≤ − 1. Proteins detected exclusively in one experimental group were also considered DAPs when meeting the statistical significance threshold (*P*-value < 0.001).

PCA was performed by SRplot platform [16] to assess global trends and variation in proteomics data, and to visualize the data set in a two-dimensional space, to reduce the dimensionality, while maximizing the discriminatory power among classes. Normalized quantitative data were used as input for the analysis. The variances explained by each principal component were reported, and graphical representations were generated.

To identify the biological processes (BP), DAPs were subjected to an enrichment analysis with terms from the Gene Ontology annotation and categorized in the class of “Biological Processes”, using the Database for Annotation, Visualization, and Integrated Discovery (DAVID 2021 Knowledgebase, v2023q4) [17, 18]. For gene ontology analysis using DAVID, the *P*-value < 0.05 was considered.

### Histone extraction

Histones were extracted from 20 mg of endometrial tissue. Tissues were macerated into small pieces, resuspended in PBS containing a protease inhibitor cocktail (A32953, Thermo Fisher Scientific, Lithuania), and disrupted using a Tissue Lyser (Qiagen), followed by centrifugation. The supernatant was discarded, and the pellet was used to extract histones, using the Histone Extraction Kit – Rapid/Ultra-pure (ab221031, Abcam), according to the manufacturer’s instructions for standard extraction. After histone content determination using the Bradford assay, it was stored at -80 °C for further Western Blot analysis.

### Western blot analysis

Western blot analysis was used to quantify protein abundance of histone H2A (Ref. PA5-17600; Invitrogen) in category IIA endometria (*n* = 6), category IIB endometria (*n* = 6), category IIA-E endometria (*n* = 4) and category IIB-E endometria (*n* = 5). The histone extracts were separated by SDS-PAGE (12.5% acrylamide gel) and transferred to 0.45 μm Amersham polyvinylidene difluoride membranes (10600023, Amersham™ Hybond™, Georgia), which were then incubated overnight at 4 °C with the H2A antibody, diluted 1:1,000. The incubation with the secondary antibody was performed for 1.5 h at room temperature with the polyclonal Goat anti-Rabbit Immunoglobulins/HRP antibody, at a dilution of 1:10,000 (P0448; Palex, Spain). Chemiluminescent detection was achieved by incubating the membrane with SuperSignal™ West Pico Plus Chemiluminescent Substrate (34580; Thermo Fisher Scientific, Lithuania). Images were acquired using the ChemiDoc MP system and Image Lab 4.1 software XRS + (BioRad, Hercules, Canada). Due to the low total histone protein concentrations, the target protein expression was normalized using the No-Stain™ Protein Labeling Reagent Kit (A44449, Thermo Fisher Scientific, Lithuania). For each sample, the pixel counts of the H2A histone were divided by the respective pixel counts of the total extracted histones and analyzed by computerized densitometry using Image Lab 4.1 software XRS + (BioRad, Hercules, Canada).

### Statistical analysis

For Western blot analyses, statistical analysis was performed using the GraphPad Prism version 8 (GraphPad Software, San Diego, CA, USA). Western blot data are presented in arbitrary units. The Gaussian distribution was tested using the Shapiro-Wilk normality test. The differences between two independent groups were compared using the Mann–Whitney U test. A *p*-value < 0.05 was considered statistically significant.

## Results

### Endometrium proteome of mares with endometritis and endometrial fibrosis

**Fig. 1:**
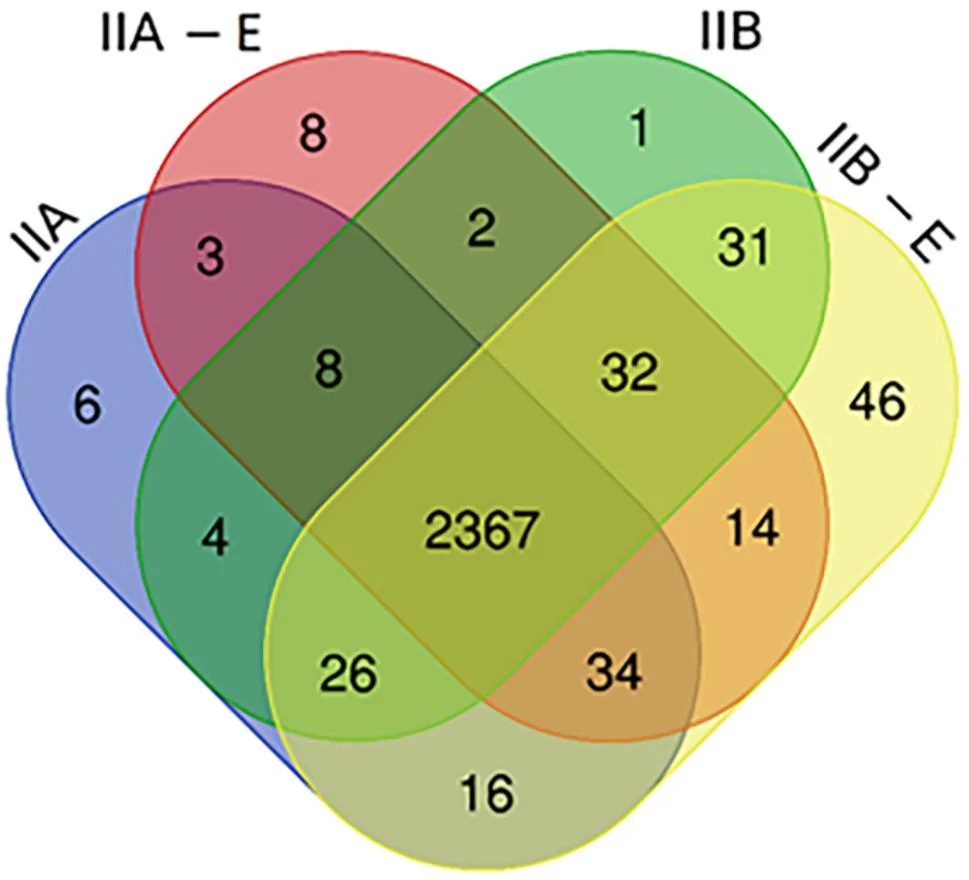
Venn diagram showing the exclusive and common proteins in the endometria from IIA, IIA-E, IIB and IIB-E categories; Diagram created based on content available at: https://bioinformatics.psb.ugent.be/webtools/Venn/ (accessed August 6, 2025)

A total of 2,464 proteins from category IIA endometria, 2,468 proteins from IIA-E endometria, 2,471 proteins from IIB endometria, and 2,566 proteins from IIB-E endometria were quantified. Venn diagrams were used to identify the number of proteins specific to each group, and proteins common to the different groups (Fig. 1). A total of 2,367 proteins were common to all endometria, while six proteins were found exclusively in category IIA, eight proteins were specific to category IIA-E, one protein was specific to category IIB, and 46 proteins were specific to category IIB-E endometria (Fig. 1) (Supplementary Table 1). Fourteen proteins were common to endometria with endometritis regardless of the category (IIA-E and IIB-E), which were not detected in endometria without endometritis. They include five proteins related to inflammation: integrin beta 2, serum amyloid A (SAA1), myosin 1B, diaphanous related formin 1, and sulfhydryl oxidase.

### Changes in the endometrial proteome associated with endometritis

**Fig. 2:**
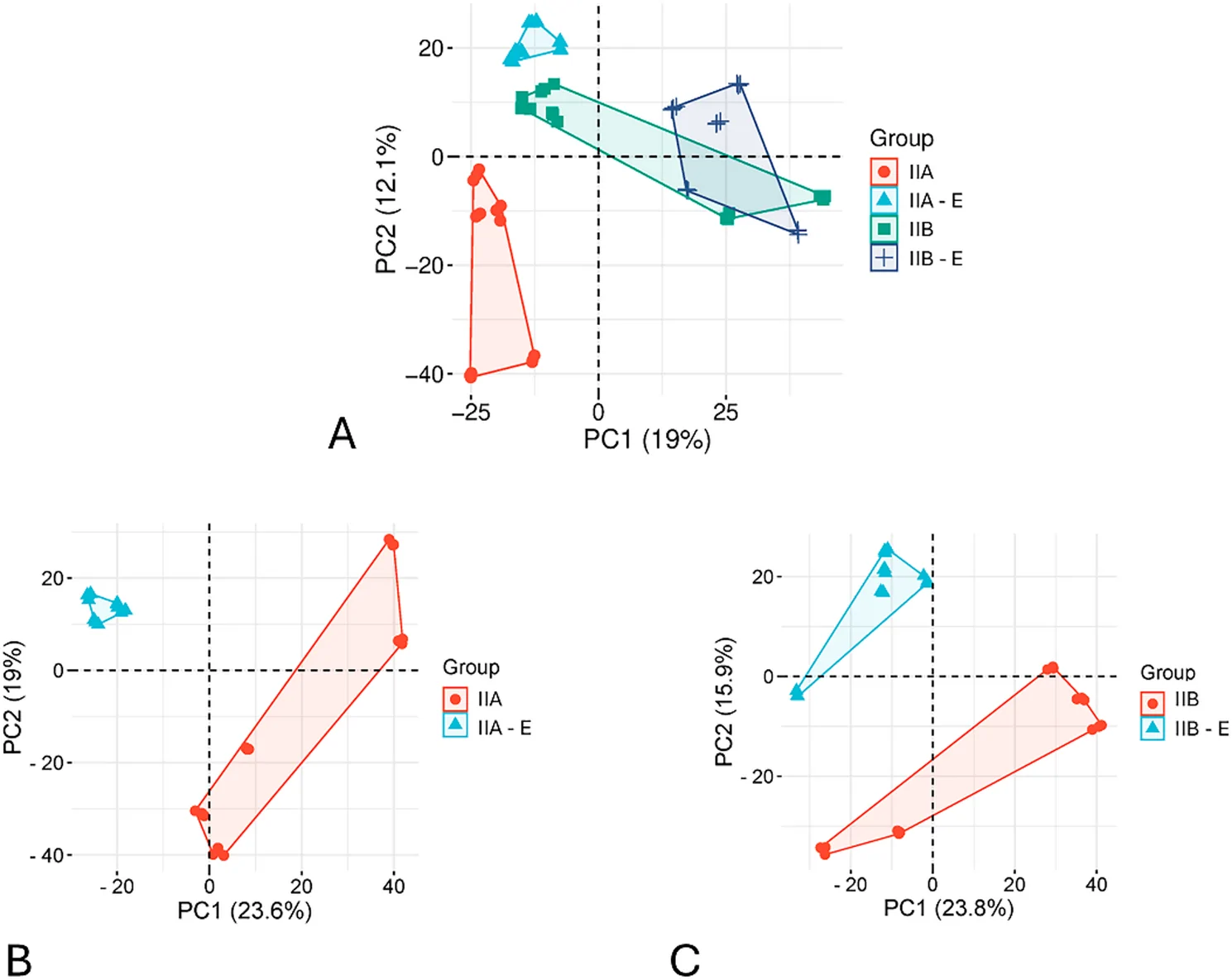
Principal component analysis (PCA) of proteomic data from endometrium with and without endometritis; (**A**) PCA including all four groups (**B**) PCA of the category IIA vs. IIA-E endometria; (**C**) PCA of category IIB vs. IIB-E endometria

To assess the changes in the endometrium proteome associated with endometritis, the proteome was compared within the same category, with and without endometritis. The PCA showed a clear clustering of individuals associated with group in the endometria from categories IIA (IIA vs. IIA-E; Fig. 2A) and IIB (IIB vs. IIB-E; Fig. 2B). The projection of data on the two principal components explains around 43% (IIA vs. IIA-E) and 40% (IIB vs. IIB-E) of the variance.

**Fig. 3:**
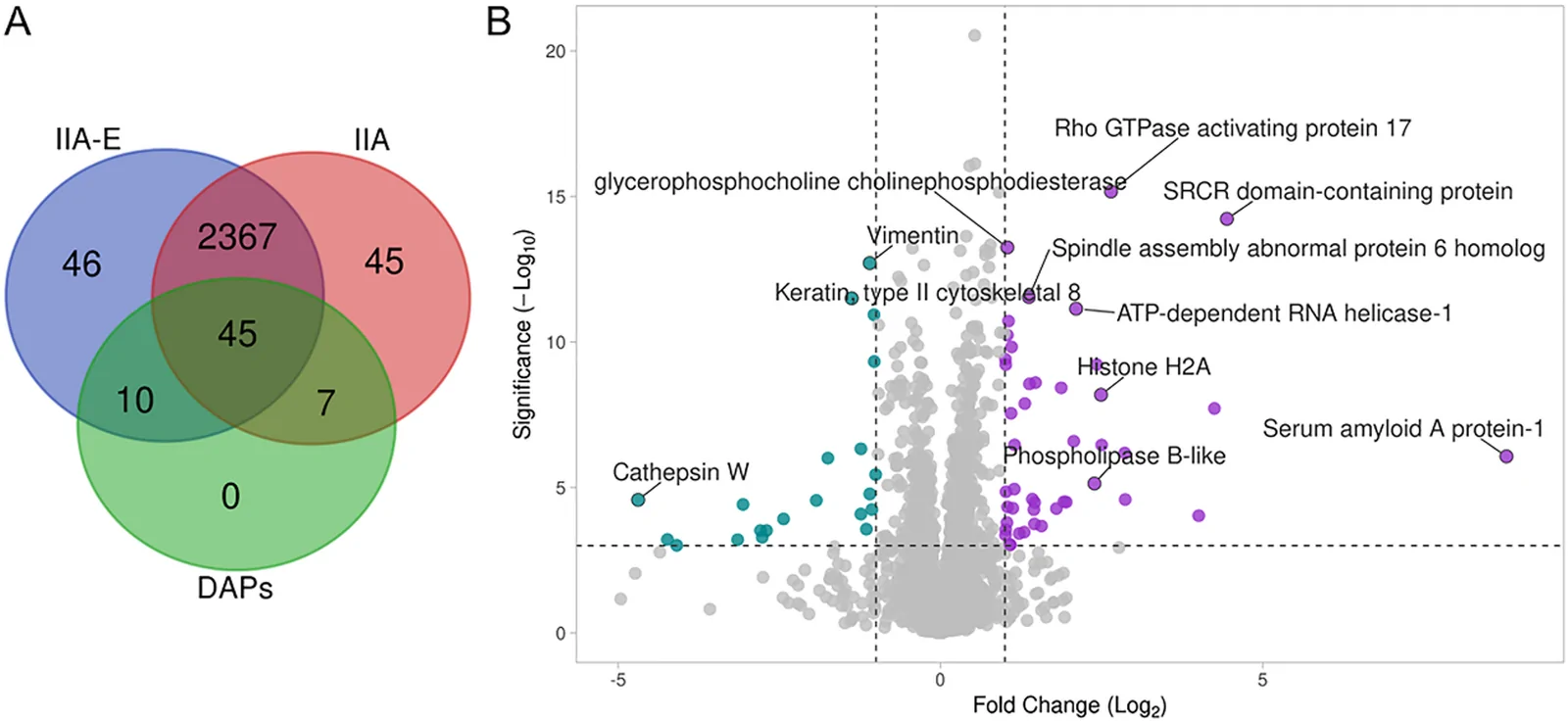
**A** Venn diagram representing the DAPs found in category IIA-E endometria, when compared with endometria from category IIA; (**B**) Volcano plot representing the DAPs upregulated and downregulated in category IIA-E endometria, compared to category IIA endometria; Diagram created based on content available at: https://bioinformatics.psb.ugent.be/webtools/Venn/ (accessed May 8, 2026)

A total of 2,412 proteins were found in both endometria IIA and IIA-E, of which 45 were differentially abundant (P-value < 0.001; -1 ≥ Log2 Fold Change ≥ 1). Of the differentially abundant proteins (DAPs), 7 related to apoptosis and transport were specific to category IIA, while 10 DAPs related to cellular oxidant detoxification, system development and Wnt signalling pathway were only found in the endometrium of category IIA-E (Fig. 3A). Of the 62 DAPs found in category IIA-E, 41 were upregulated and 21 were downregulated [19] (Fig. 3B).

**Fig. 4:**
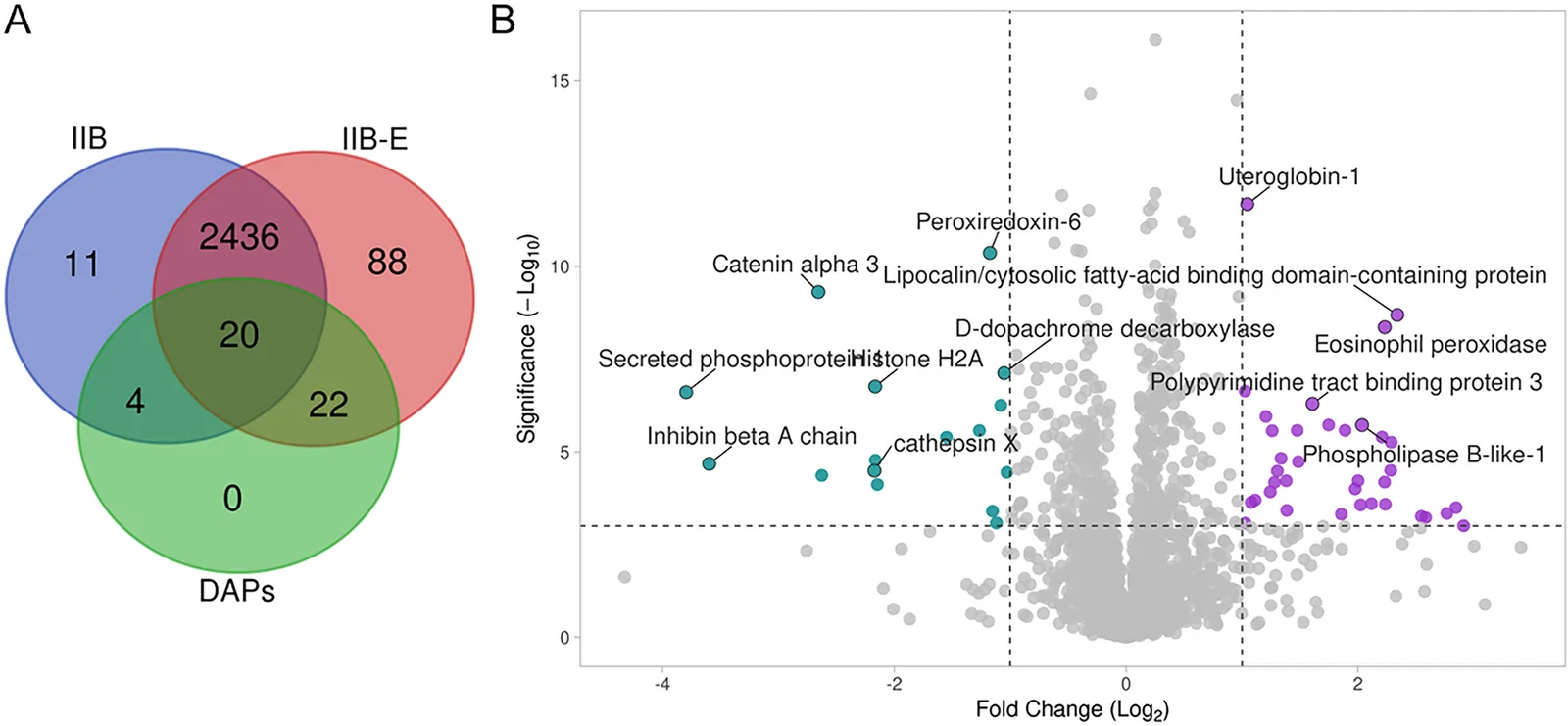
**A** Venn diagram representing the DAPs found in the category IIB-E compared to category IIB endometria; (**B**) Volcano plot representing the upregulated and downregulated DAPs in category IIB-E endometria, compared to category IIB endometria; Diagram created based on content available at: https://bioinformatics.psb.ugent.be/webtools/Venn/ (accessed May 8, 2026)

A total of 2,456 proteins were found in both endometria IIB and IIB-E, of which 20 were differentially abundant. Four DAPs related to cell adhesion and regulation of gene expression were specific to category IIB endometrium, while 22 DAPs related to DNA replication, defense response, innate immune response and inflammatory response, were only found in category IIB-E endometrium (Fig. 4A). Of the 46 DAPs found in IIB-E endometria, 33 were upregulated and 13 were downregulated [19] (Fig. 4B).

The top 25 DAPs in endometria with endometritis include proteins that have been previously reported in equid endometritis [5, 11, 12, 20]. These include the SAA1, isoforms of serpins, histone H2A, secreted phosphoprotein 1 (SPP1), azurocidin 1 (AZU1), isoforms of lipocalins and isoforms of myosin (Tables 1 and 2), which were also differentially abundant in the uterine fluid of mares and serum of jennies with endometritis [5, 11, 12, 20]. Additionally, our study identified other proteins related to endometritis for the first time. These include the hepsin, different types of phospholipases and eosinophil peroxidase (EPO) (Tables 1 and 2).

**Table 1:** The top twenty-five dysregulated proteins in category IIA-E, compared with category IIA endometria

Name	Change	Fold change (log2)	Significance	Manhattan distance
Serum amyloid A protein	Increased	8.781	6.072	14.853
Cathepsin W	Decreased	-4.690	4.576	9.266
SRCR domain-containing protein	Increased	4.443	14.231	18.674
Inhibin beta A chain	Increased	4-250	7.717	11.967
Calcineurin B homologous protein 1	Decreased	-4.236	3.215	7.451
Retinol binding protein 3	Decreased	-4.092	3.013	7.105
Nucleoredoxin	Increased	4.006	4.033	8.039
RNA-binding protein 47	Decreased	-3.147	3.208	6.355
Ig-like domain-containing protein	Decreased	-3.064	4.419	7.482
Mucin 6, oligomeric mucus/gel-forming	Increased	2.866	4.588	7.454
RTF1 homolog, Paf1/RNA polymerase II complex component	Increased	2.860	6.187	9.046
Short/branched chain specific acyl-CoA dehydrogenase, mitochondrial	Decreased	-2.792	3.523	6.315
Protein phosphatase, Mg2+/Mn2 + dependent 1 F	Decreased	-2.765	3.292	6.058
Zinc finger CCCH domain-containing protein 14	Decreased	-2.699	3.523	6.222
Rho GTPase activating protein 17	Increased	2.645	15.171	17.816
Serpin domain-containing protein	Increased	2.501	6.461	8.962
Histone H2A	Increased	2.491	8.189	10.679
Ig-like domain-containing protein	Decreased	-2.434	3.921	6.355
Serpin family A member 1	Increased	2.426	9.224	11.650
Phospholipase B-like	Increased	2.389	5.138	7.527
ATP-dependent RNA helicase	Increased	2.104	11.144	13.248
Peroxidasin	Increased	2.067	6.592	8.659
EF-hand domain-containing protein	Increased	1.950	4.500	6.450
Peptidase S1 domain-containing protein	Decreased	-1.926	4.557	6.483
Uracil-DNA glycosylase	Increased	1.914	4.511	6.425

**Table 2:** The top twenty-five dysregulated proteins in category IIB-E compared with category IIB endometria

Name	Change	Fold change (log2)	Significance	Manhattan distance
Secreted phosphoprotein 1	Decreased	-3.796	6.609	10.404
Inhibin beta A chain	Decreased	-3.597	4.676	8.273
Resistin	Increased	2.912	3.004	5.917
2’-5’ oligoadenylate synthase	Increased	2.846	3.495	6.341
Azurocidin 1	Increased	2.767	3.337	6.105
Catenin alpha 3	Decreased	-2.655	9.311	11.966
Kinesin family member 5 C	Increased	2.584	3.222	5.806
Ig-like domain-containing protein	Increased	2.548	3.260	5.807
Lipocalin/cytosolic fatty-acid binding domain-containing protein	Increased	2.339	8.693	11.032
Myosin IB	Increased	2.286	5.258	7.544
RNA-binding protein 47	Increased	2.282	4.499	6.782
Interferon-induced GTP-binding protein Mx2	Increased	2.236	3.585	5.821
Eosinophil peroxidase	Increased	2.231	8.361	10.591
protein-serine/threonine phosphatase	Increased	2.230	4.185	6.415
DNA replication licensing factor MCM6	Increased	2.208	5.399	7.607
cathepsin X	Decreased	-2.171	4.490	6.661
Histone H2A	Decreased	-2.165	6.760	8.925
Hepsin	Decreased	-2.164	4.774	6.937
Lipocalin 2	Decreased	-2.146	4.119	6.265
Adaptor related protein complex 3 subunit sigma 1	Increased	2.117	3.602	5.719
Phospholipase B-like	Increased	2.036	5.720	7.756
Uncharacterized protein	Increased	2.023	3.569	5.592
Phospholipase D family member 4	Increased	2.001	4.221	6.223
Phospholipase A1 member A	Increased	1.976	4.000	5.976
Serpin domain-containing protein	Increased	1.889	5.579	7.468

**Fig. 5:**
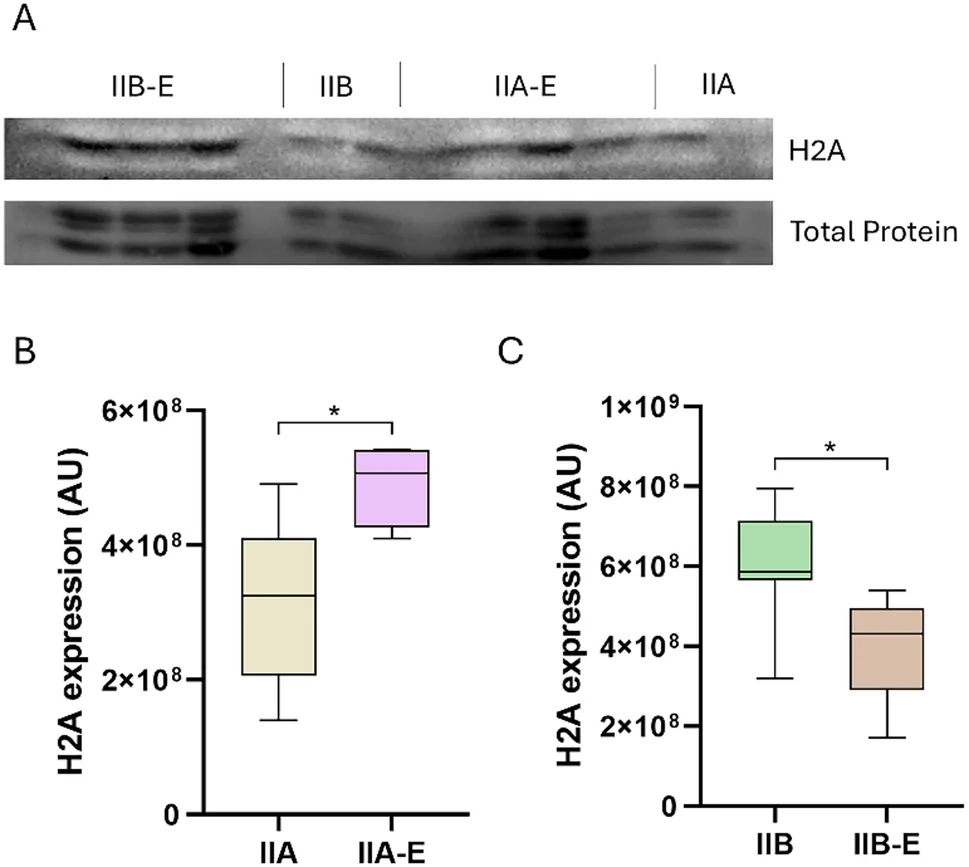
Relative protein abundance of histone H2A, quantified by Westen Blot; (**A**) Western blots for histone H2A and respective total histones for normalization; (**B**-**C**) Box plots represent the interquartile range (Q1–Q3), with the horizontal line indicating the median and the whiskers representing the minimum and maximum values. Statistical significance was assessed using Man-Whitney test; *p < 0.05

Of these 25 top proteins, only seven were common to both endometria with endometritis (IIA-E, IIB-E), regardless of the level of fibrosis (mild vs. moderate). These include the cathepsins, Inhibin beta A chain, RNA-binding protein 47, histone H2A, phospholipase B-like, serpin domain-containing, and Ig-like domain-containing protein. However, the abundance (downregulation or upregulation) of inhibin beta A chain, RNA-binding protein 47, histone H2A, and Ig-like domain-containing protein, in endometritis depends on the degree of fibrosis. Specifically, the western blot analysis confirmed the protein abundance of histone H2A identified by proteomics, which showed that histone H2A protein abundance was significantly increased (*P* < 0.05) in category IIA-E (Fig. 5A and B), when compared to category IIA, and significantly decreased (*P*-value < 0.05) in category IIB-E when compared with category IIB (Fig. 5A and C).

### Functional analysis of DAPs

**Fig. 6:**
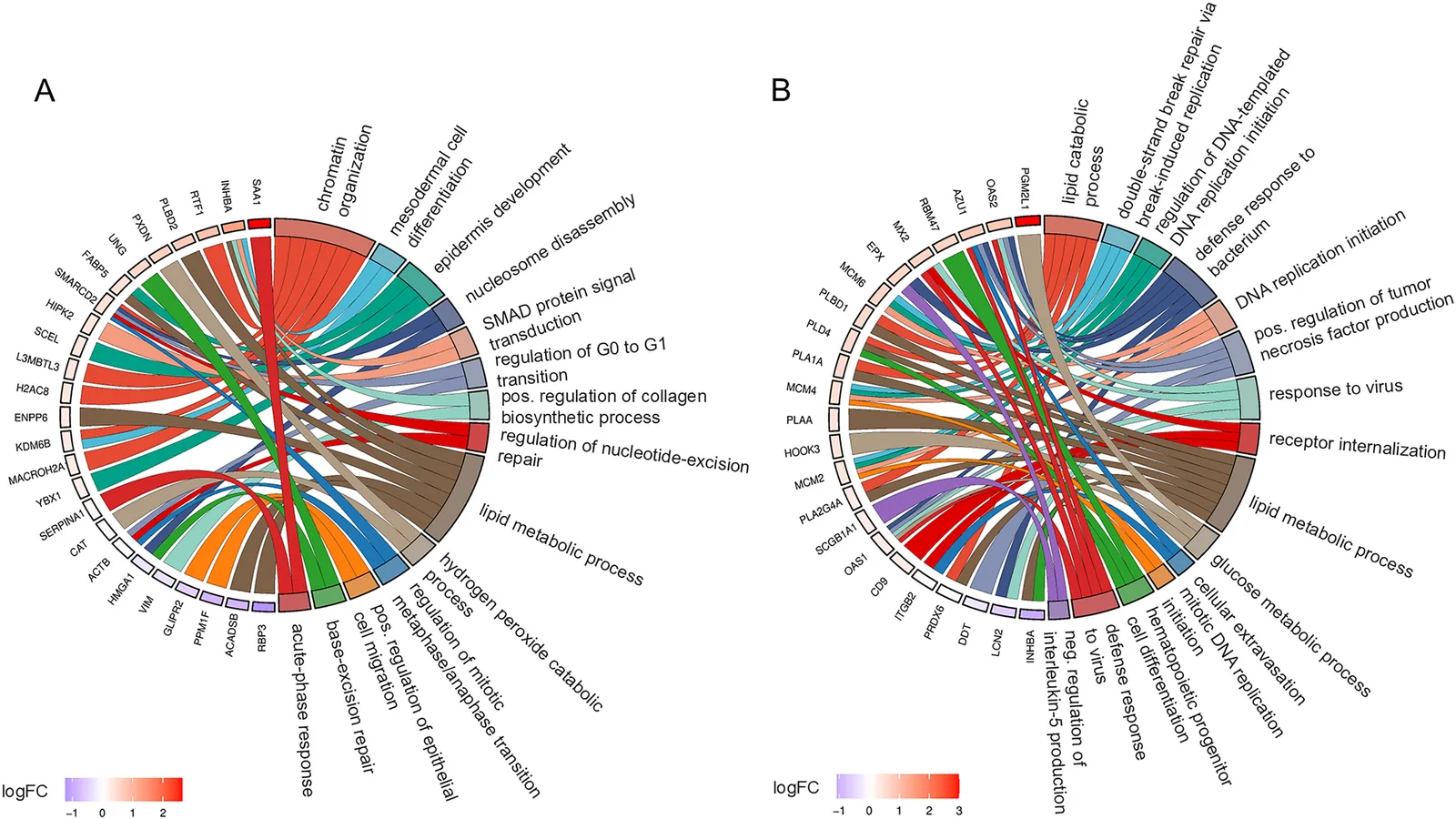
**A** The fourteen significantly enriched biological processes enriched by differently abundant proteins (DAPs) detected in IIA-E endometria, compared to IIA endometria; **B** The TOP fifteen significantly enriched biological processes, enriched by DAPs detected in IIB-E endometria, compared to IIB endometria

Fourteen significant BP (*p*-value < 0.05) were changed in category IIA-E compared with IIA (Fig. 6A). The DAPs in IIA-E were enriched in BP related to chromatin remodelling (chromatin organization, nucleosome disassembly, regulation of nucleotide-excision repair and base-excision repair), cell cycle regulation (regulation of G0 to G1 transition, and regulation of mitotic metaphase/anaphase transition), inflammatory response (acute-phase response), and tissue differentiation and remodelling (mesodermal cell differentiation, SMAD protein signal transduction, positive regulation of collagen biosynthetic process, and positive regulation of epithelial cell migration) (Fig. 6A).

The DAPs in IIB-E, when compared with category IIB endometria, were enriched in 35 significant BP (*p*-value < 0.05). The TOP 15 BP included terms related to DNA replication (double-strand break repair via break-induced replication, regulation of DNA-templated DNA replication initiation, DNA replication initiation and mitotic DNA replication initiation), immune/inflammatory response (defense response to bacterium, positive regulation of tumor necrosis factor production, response to virus, defense response to virus, cellular extravasation, and negative regulation of interleukin-5 production), and metabolic regulation (lipid catabolic process, lipid metabolic process and glucose metabolic process) (Fig. 6B).

Overall, BPs related to immune/inflammatory response, DNA-related processes and cell cycle, were altered in both endometria associated with endometritis (IIA-E and IIB-E), although the proteins involved depended on the category/extent of fibrosis (Fig. 6A and B).

## Discussion

Endometritis and endometrosis are among the most significant reproductive disorders affecting mares, with major implications for equine fertility. Although these conditions have been extensively described histopathologically, the underlying molecular mechanisms remain poorly elucidated. In this context, proteomic analyses represent a promising approach to better understand the complex molecular landscape underlying these uterine pathologies. The identification of DAPs provides crucial insights into disease progression and uterine dysfunction, while highlighting potential protein biomarkers for early and more accurate diagnosis. Furthermore, unravelling the molecular pathways implicated may open new perspectives for targeted therapeutic interventions and improved reproductive management strategies.

This study performed a proteomic analysis of endometrial tissue from mares, with and without endometritis, when associated with fibrosis. The objective was to characterize the proteomic alterations associated with endometritis and to explore how the degree of endometrial fibrosis influences the molecular response to uterine inflammation. Endometria with endometritis (IIA-E vs. IIA; IIB-E vs. IIB) exhibited a significantly higher abundance of proteins, suggesting changes in the uterine milieu that, ultimately, could impact the establishment and maintenance of pregnancy. Around 91% (*n* = 2,367) of the proteins quantified in this study were common to all endometria, which exhibited either minor or moderate histopathological changes. Nevertheless, only 2.7% (*n* = 64) of these common proteins are differentially abundant. These include proteins, such as serpins (serpin family A member 1 and serpin domain-containing), histone H2A, and different types of phospholipases (phospholipase A1A, phospholipase A2, phospholipase B-like and phospholipase D4). Serpin proteins are a family of protease inhibitors involved in many cellular processes, including the regulation of inflammation and tissue remodelling [21]. Previous studies identified lower abundances of serpin family C member 1 in the uterine fluid of mares with endometritis or fibrotic endometrial degeneration, compared to healthy ones [11]. Serpin family A member 3 was also downregulated in the serum of jennies with infectious endometritis [20]. In our study, serpin family A member 1 (SERPINA1) and a serpin domain-containing protein were upregulated in endometritis in IIA category (IIA-E vs. IIA). Similarly, serpin domain-containing protein was upregulated in endometritis in IIB category (IIB-E vs. IIB). Serpins, including SERPINA1, are serine protease inhibitors that can inhibit the neutrophil serine proteases and other serine proteases. In fact, since serine proteases are key contributors to inflammation, the regulation of their activity by serpin proteins is crucial to prevent excessive inflammation and tissue damage [22–24]. Therefore, the upregulation of serpins in endometritis should be a mechanism to counteract the activity of the serine proteases, to maintain homeostasis and avoid excessive inflammation. Similarly, four phospholipases (phospholipase A1A, phospholipase A2, phospholipase B-like and phospholipase D4) were upregulated in endometria IIB-E, compared to IIB, while only one (phospholipase B-like) was upregulated in endometria IIA-E, compared to IIA. Phospholipases are enzymes that hydrolyze phospholipids and are classified into four main types (A, B, C, and D), depending on the ester linkage that is cleaved within the phospholipid molecule [25]. The lipid mediators generated by these enzymes regulate several processes, such as the cytoskeletal dynamics, homeostasis, nutrient acquisition, inflammation and host defence [25]. Specifically, phospholipase A1A can promote inflammation by generating lysophosphatidylserine (LysoPS) and activating LysoPS receptors on myeloid cells [26]. Phospholipase A2 regulates the release of arachidonic acid from phospholipids, thereby regulating the availability of this precursor for the synthesis of inflammatory mediators like leukotrienes and platelet activating factor-acetylhydrolases [27]. In mare endometritis, treatment with anti-inflammatory drugs that modulate the phospholipase A2 pathway has been recommended for mares susceptible to endometritis to prevent excessive inflammation [28]. Therefore, the upregulation of phospholipases in endometria with endometritis reflects the presence of a higher level of inflammation in these endometria. In particular, the upregulation of several phospholipases in IIB-E endometria reflects its higher inflammatory state. In contrast, H2A histone showed an expression pattern associated with the inflammatory state of the endometrium. Histones can regulate gene transcription through DNA binding, influencing the chromatin accessibility and, consequently, cellular states. Therefore, histone H2A modulates the inflammatory response by regulating the transcription of pro-inflammatory genes [29–32]. Our proteomic and Western blot analyses showed increased histone H2A protein abundance in endometritis in IIA category endometrium (IIA-E vs. IIA). By contrast, histone H2A protein abundance decreased in endometria with endometritis associated with endometrosis (IIB-E vs. IIB). A higher protein abundance of H2A was also reported in the uterine fluid of mares with endometritis, compared to healthy ones, although the mares’ endometria category was not addressed [5]. Our data suggest that the expression of histone H2A is associated with the stage and progress of the inflammatory process. Therefore, this protein might promote inflammation in IIA-E endometria, as a primary response to the inflammatory stimuli. In IIB-E, with more prolonged pathological processes, the expression of H2A is possibly repressed to avoid excessive inflammation and tissue damage. Overall, this revealed that the response to endometritis might be influenced by the endometrium milieu, such as the already activated immune response and/or the degree of fibrosis.

Other proteins showed a higher abundance in endometrium with endometritis, only when associated with endometrosis (IIB-E). These include the azurocidin 1 (AZU1) protein, eosinophil peroxidase (EPO), and secreted phosphoprotein 1 (SPP1). AZU1 is present in the azurophilic granules and secretory vesicles within neutrophils, and it is released in response to bacterial antigens, at an early stage of infection, therefore, playing a relevant role during the initiation of the immune response [12, 33, 34]. This protein is involved in the recruitment and activation of monocytes and macrophages, promoting the release of cytokines and bacterial phagocytosis, with its activity mediated by β2-integrins [33]. Therefore, the upregulation of this protein in endometria with infectious endometritis is consistent with a protective immune response against bacterial infection. Similarly, EPO, an enzyme present within eosinophil granules and released as eosinophils degranulate, was upregulated only in the endometria with endometritis associated with endometrosis, suggesting a more severe and persistent inflammation involving the recruitment and activation of eosinophils. While eosinophils play a role in maintaining tissue homeostasis, their accumulation in tissues has been associated with several diseases. Under these conditions, activated eosinophils release inflammatory mediators, including proinflammatory lipid mediators, cytokines, and free oxygen radicals. These mediators contribute to the onset and maintenance of tissue inflammation [35]. EPO has a dual activity, involving the production of reactive oxygen species that damage bacterial cells, as part of the host defence mechanism, but also generates oxidative stress, causing tissue damage [36]. In the mare, an increased eosinophil count in the endometrium, exceeding the physiological range, defined as eosinophilic endometritis, was associated with other endometrial diseases, including endometrosis [37]. This eosinophilic infiltration was also associated with the presence of neutrophils. Similarly, in our study, endometritis was also associated with fibrosis (IIB-E), which possibly justifies a higher eosinophil infiltrate and the upregulation of EPO in these endometria. Consequently, the association of these endometrial pathologies would have a greater impact on fertility. In agreement with this, the SPP1, also known as osteopontin, was the most altered protein in category IIB-E endometria (downregulated), when compared to category IIB. This protein interacts with integrin receptors on the endometrium and in the embryo trophectoderm, playing a crucial role in the embryonic adhesion during implantation in both the ewe and the sow [38]. This protein is also expressed in the equine endometrium and embryo at day 16 of pregnancy [39], and it has been suggested that a reduction in SPP1 in uterine fluid might contribute to impaired uterine receptivity in mares with endometritis [11]. Therefore, the downregulation of SPP1, specifically in endometria with endometritis associated with endometrosis (IIB-E), suggests that the endometrial receptivity is compromised in these endometria.

Our study showed that DAPs in endometritis (IIA-E vs. IIA; and IIB-E vs. IIB) were significantly enriched in BP related to immune and inflammatory response, DNA-related processes and cell cycle. During inflammation or infection, tissue repair and regeneration are associated with increased cellular proliferation, activation of DNA replication, chromatin remodelling, and cell cycle-related pathways. In addition, inflammatory processes and oxidative stress can induce DNA damage, leading to the activation of DNA repair mechanisms [40, 41]. The DNA-related and cell cycle-related biological processes in equine endometritis may therefore reflect enhanced proliferation and repair activity in response to tissue injury and inflammation. As category IIA-E endometrium is at an early stage of the fibrosis mechanism, the BP related to tissue differentiation and remodelling (SMAD protein signal transduction, positive regulation of collagen biosynthetic process, and positive regulation of epithelial cell migration) and cell cycle regulation (regulation of G0 to G1 transition and regulation of mitotic metaphase/anaphase transition) may reflect the endometrium attempt to compensate for structural damage caused by mild fibrosis and endometritis. Most of the BP in IIB-E endometria were associated with DNA replication (double-strand break repair via break-induced replication, regulation of DNA-templated DNA replication initiation, DNA replication initiation and mitotic DNA replication initiation) and immune/inflammatory response (defense response to bacterium, positive regulation of tumor necrosis factor production, response to virus, defense response to virus, cellular extravasation, and negative regulation of interleukin-5 production). In IIB-E endometria, these BP may reflect increased cellular turnover, activation of DNA repair mechanisms, and persistent inflammatory activity associated with more severe endometrial damage and fibrosis (endometritis associated with moderate endometrosis). Such findings suggest enhanced proliferative and repair response of endometrial and immune cells striving to maintain tissue homeostasis under chronic inflammatory conditions.

Despite being an innovative study, our proteomic data analysis had an important limitation. It defined minimal criteria for protein identification (the presence of at least two unique peptides per biological replicate and valid LFQ values in at least three biological replicates per group) to reduce false positives and noise and improve reliability. Nevertheless, it might also exclude low-abundance and condition-specific proteins.

## Conclusions

This pioneering study showed that the presence of endometrosis may influence the expression of specific proteins and molecular processes involved in mare endometritis. Specifically, the abundance of histone H2A is dependent on the level of fibrosis. Several proteins specifically dysregulated in endometrium IIB-E (azurocidin 1, eosinophil peroxidase, and secreted phosphoprotein 1) may also reflect an attempt to counteract the severe structural damage caused by endometrosis and endometritis. This suggests that the endometritis-activated processes are influenced by the endometrium milieu. Nevertheless, other inflammation-related proteins (serpins and several phospholipases) were specifically linked to endometritis, as they were upregulated in these endometria, regardless of the degree of fibrosis. The DAPs in these endometria (IIA-E; IIB-E) were mostly enriched in BP related to immune and inflammatory response, DNA-related processes and cell cycle, which likely represent core mechanisms of mare endometritis.

## Supplementary Information

Supplementary Material 1.

## Data Availability

The mass spectrometry proteomics data have been deposited to the ProteomeXchange Consortium via the PRIDE partner repository with the dataset identifier PXD070769.

## Abbreviations

AZU1: Azurocidin 1
BP: Biological Processes
DAPs: Differentially Abundant Proteins
DAVID: Data Annotation, Visualization, and Integrated Discovery
EPO: Eosinophil Peroxidase
LC-MS/MS: Liquid Chromatography-tandem Mass Spectrometry
LQF: Valid Quantification Values
LysoPS: Lysophosphatidylserine
PCA: Principal Component Analysis
SAA1: Serum Amyloid A
SERPINA1: Serpin family A member 1
SPP1: Secreted Phosphoprotein 1
SWATH: Sequential Window Acquisition of all Theoretical fragment ion spectra
